# Rapid flood and damage mapping using synthetic aperture radar in response to Typhoon Hagibis, Japan

**DOI:** 10.1038/s41597-020-0443-5

**Published:** 2020-03-25

**Authors:** Cheryl W. J. Tay, Sang-Ho Yun, Shi Tong Chin, Alok Bhardwaj, Jungkyo Jung, Emma M. Hill

**Affiliations:** 1 Asian School of the Environment, Nanyang Technological University, Singapore, Singapore; 2 Jet Propulsion Laboratory, California Institute of Technology, Pasadena, CA USA; 3 Earth Observatory of Singapore, Nanyang Technological University, Singapore, Singapore

**Keywords:** Natural hazards, Geophysics

## Abstract

During the aftermath of Typhoon Hagibis, we made flood and damage proxy maps, rapidly derived from synthetic aperture radar (SAR) data using change detection approaches. The maps have large spatial coverage over the Tokyo, Fukushima, Ibaraki, Iwate, and Nagano prefectures of Japan. The maps are also largely in agreement with various validation sources including aerial imagery, optical imagery and news sources. Apart from visual maps, we provide flood and damage extents in various formats compatible with geographic information system (GIS) applications. The data may potentially be used for applications such as typhoon risk modelling, investigating spatial correlations of typhoon impacts, and comparing alternative flood or damage mapping techniques.

## Abstract (summary)

**Table:** 

Measurement(s)	damage • flood
Technology Type(s)	synthetic aperture radar
Factor Type(s)	geographic location
Sample Characteristic - Environment	cyclone
Sample Characteristic - Location	Japan

Machine-accessible metadata file describing the reported data: 10.6084/m9.figshare.11968443

## Background & Summary

Typhoon Habigis made landfall at the Izu Peninsula of Shizuoka Prefecture, Japan on 12 October 2019, UTC 08:30 as a Category 2-equivalent on the Saffir Simpson scale^1^. It was one of the strongest typhoons of the century to hit Japan, and had one of the fastest intensifications from a Category 1 to 5 status in less than a day. Reports reveal widespread impacts and at least 80 deaths, 135,000 people affected, 68,000 houses inundated and 10,000 buildings damaged^1,2^.

In response to the event, we mapped flood and damage extents across Japan over a week after the typhoon’s landfall to potentially support stakeholders in the region in assessing impacts and planning disaster relief efforts. The maps are derived from data acquired from SAR, a form of remote sensing. SAR is particularly helpful compared to other modes of remote sensing as its radar transmission in the microwave spectrum is independent of day-and-night visibility and weather conditions, such as persistent rain and cloud cover observed during typhoons. To identify flood and damage from SAR, we used change detection approaches where data acquired before and during an event are compared to detect land surface changes.

With the frequent six-day revisit of the Copernicus Sentinel-1 satellites operated by the European Space Agency, and ALOS-2 tasking efforts coordinated by the Japan Aerospace Exploration Agency and Sentinel Asia, timely SAR data were acquired following Typhoon Hagibis’ landfall. Rapid mapping with SAR data was facilitated by the Advanced Rapid Imaging and Analysis Singapore (ARIA-SG) system at the Earth Observatory of Singapore. This is an automated and scalable cloud-based SAR processing system for near real-time monitoring and rapid response for natural disasters. It is originally cloned from the ARIA system developed by the Jet Propulsion Laboratory and California Institute of Technology. Finally, four flood proxy maps (FPMs) and one damage proxy map (DPM) were disseminated through Sentinel Asia, a network comprising satellite data providers, analysis nodes and government agencies to support disaster management activity in the Asia-Pacific region^3^.

The paper is organized as follows: (1) principles of the SAR-based change detection techniques are explained in Methods; (2) maps are shown in Data Records; (3) and accuracies and uncertainties of the maps are discussed in Technical Validation for notes on usage of the maps. Due to the large spatial coverage of SAR data, the maps are most useful for providing relative spatial distribution information on the typhoon impacts. Potential usages of the data include typhoon risk modelling, investigation of spatial correlations of typhoon impacts, and comparisons with alternative flood or damage mapping techniques.

## Methods

### Flood proxy map

We obtained pairs of Sentinel-1 and ALOS-2 scenes (Table 1). Each pair consists of a scene acquired before, and another acquired after Typhoon Hagibis’ landfall. We selected each pre-event scene with acquisition time within a period of minimal precipitation, such that a pair can be used to detect floods brought by the typhoon using a change detection approach.

**Table 1:** Parameters of SAR data and threshold LAR and COD values used for flood and damage mapping respectively.

Coverage	Map	Sensor	Mode, Polarization	Orbit Direction	Pre-event Scene Date(s)	Co-event Scene Date	Threshold Type	Negative Threshold	Positive Threshold
Tokyo	FPM	Sentinel-1	IW, VV/VH	D	24 Sep 2019, UTC 20:43	12 Oct 2019, UTC 20:43	LAR	−0.130	0.100
Tokyo	DPM	Sentinel-1	IW, VV/VH	D	24 Sep 2019, UTC 20:43 6 Oct 2019, UTC 20:43	12 Oct 2019, UTC 20:43	COD	NA	0.270
Fukushima	FPM	Sentinel-1	IW, VV/VH	D	24 Sep 2019, UTC 20:43	12 Oct 2019, UTC 20:43	LAR	−0.130	0.130
Ibaraki	FPM	ALOS-2	SM, HH	D	27 May 2019, UTC 03:17	14 Oct 2019, UTC 03:17	LAR	−0.070	0.200
Nagano	FPM	ALOS-2	SM, HH	D	16 Jun 2015, UTC 03:37	15 Oct 2019, UTC 03:37	LAR	−0.050	0.085

IW, SM, V, H and D refer to interferometric wide swath, stripmap, vertical, horizontal and descending data respectively.

Next, we pre-processed Sentinel-1 single look complex (SLC) images in the ARIA-SG system using the InSAR Scientific Computing Environment (ISCE) processor^4^. Each co-event scene was co-registered to its corresponding pre-event scene. Co-registration used the National Aeronautics and Space Administration Version 3.0 Shuttle Radar Topography Mission (SRTM) Global 1 arc second digital elevation model (DEM)^5^ and dense subpixel offsets computed from the cross correlation of SAR amplitudes. All SLCs were then geocoded and resampled to the DEM’s spacing of approximately 30 m. For ALOS-2, we obtained L2.1 amplitude images from Sentinel Asia. These have been orthorectified and geocoded using a DEM.

After pre-processing, we used the amplitude component of SLCs, *A* to compute the logarithmic amplitude ratio (LAR)^6^ between each pair of pre- and co-event SLCs (Eq. 1). ALOS-2 LARs were also passed through a median filter to reduce speckle noise^7^, and resampled to 25 m spacing.

1 $$LAR{=\log }_{10}\left(\frac{{A}_{co-event}}{{A}_{pre-event}}\right)$$

Typically, flood results in amplitude decrease or a negative LAR due to the specular surface of open waters as less signal is backscattered. Occasionally, amplitude increase over open water surfaces may be observed due to wind-driven capillary waves. Flood also results in amplitude increase or a positive LAR if the water body is adjacent to a semi-vertical feature, such as vegetation or buildings, due to a double bounce effect^8^. However, small amplitude changes may be attributed to noise as opposed to significant change due to actual floods.

Thus, we selected thresholds for both negative and positive LAR values to determine suitable extents of change which represent actual flooding. Thresholds were selected in consideration of the topography, and when available, known flooded places from aerial imagery from Geospatial Information Authority of Japan (GSI)^9^, optical imagery from Planet^10^ and news sources. For each FPM, all aerial imagery, optical imagery, and news sources used to guide the thresholding were acquired within 24 hours after, 5 hours after, and +/−12 hours of the SAR co-event scene acquisition respectively. All optical imagery used are 4-band PlanetScope Scenes provided in analytical mode, where the 4 bands are blue, red, green and near infrared (NIR), with values represented in digital numbers. The manual selection of thresholds using these various validation data is detailed below in Technical Validation.

Finally, we took the selected thresholds as global thresholds to apply to the whole SAR image extent. Then, all pixels with LAR values below the negative threshold or above the positive threshold were considered as flood, and flood pixels were smoothed to reduce noise. The result of thresholding is a binary image indicating flood or no flood, which was then converted into PNG, JPEG, GeoTIFF, KML and Shapefile formats for dissemination to users.

### Damage proxy map

To map damage with change detection, we obtained three Sentinel-1 SLCs (Table 1), with two scenes acquired before, and one scene acquired after the typhoon’s landfall. We used pre-event scenes which were acquired closest to the co-event scene to minimize the detection of land surface changes which occurred prior to the event. The SLCs were then pre-processed in the ARIA-SG system using ISCE algorithms^4^ similar to the Sentinel-1 flood mapping, with a resulting spacing of 30 m. For co-registration, we used the pre-event scene closest in time to the co-event scene as the reference.

We used the complex pixel value, *c* of pre-processed SLCs for the change detection analysis, where damage is inferred from a loss of coherence or decorrelation between SAR images^11^. Firstly, we computed the pre- and co-event interferometric coherences, *γ* (Eq. 2) from a pair of SLCs before the event, and another pair spanning across the event respectively.

2 $$\gamma =\frac{\left|\left\langle {c}_{1}{c}_{2}^{\ast }\right\rangle \right|}{\sqrt{\left\langle {c}_{1}{c}_{1}^{\ast }\right\rangle \left\langle {c}_{2}{c}_{2}^{\ast }\right\rangle }},\,0\le \gamma \le 1$$

The pre-event coherence represents change unrelated to the event, and was assumed to be the background value. Next, we obtained a coherence difference (COD) by subtracting ${\gamma }_{co-event}$ from ${\gamma }_{pre-event}$. Typically, human-made structures such as roads and buildings maintain high interferometric coherence, while vegetation and water surfaces show low coherence. A negative COD (or coherence gain), usually indicates surface changes occurring between the pre-event scenes and is associated with changes not related to the event. A positive COD (or coherence loss) indicates surface changes occurring between the co-event scenes spanning the event. Hence, the loss of coherence is most effective for detecting damage in built up areas caused by an event, and the types of change may include collapsed or submerged buildings and roads, and debris created by strong winds and storm surges. However, the COD is generally less effective over vegetation where coherence change may be random. Since only significant surface changes in the satellite line-of-sight can be detected, flood-induced internal building damage and cracked walls are not likely detected. We also note that greater loss in coherence generally correlates with greater severity of change, such as a fully collapsed building causing more significant coherence loss than partial collapse.

Therefore, to map damage, we determined a suitable minimum positive COD value to identify areas with significant decorrelation. Similar to flood mapping, a global threshold was manually selected based on knowledge of known damaged places from various validation sources including aerial imagery from GSI^9^ and news sources gathered in a similar way. Finally, a color map was applied to the thresholded COD to indicate increasingly significant decorrelation identified, corresponding to increasing COD values, and converted into PNG, GeoTIFF and KML formats for further use.

## Data Records

**Fig. 1:**
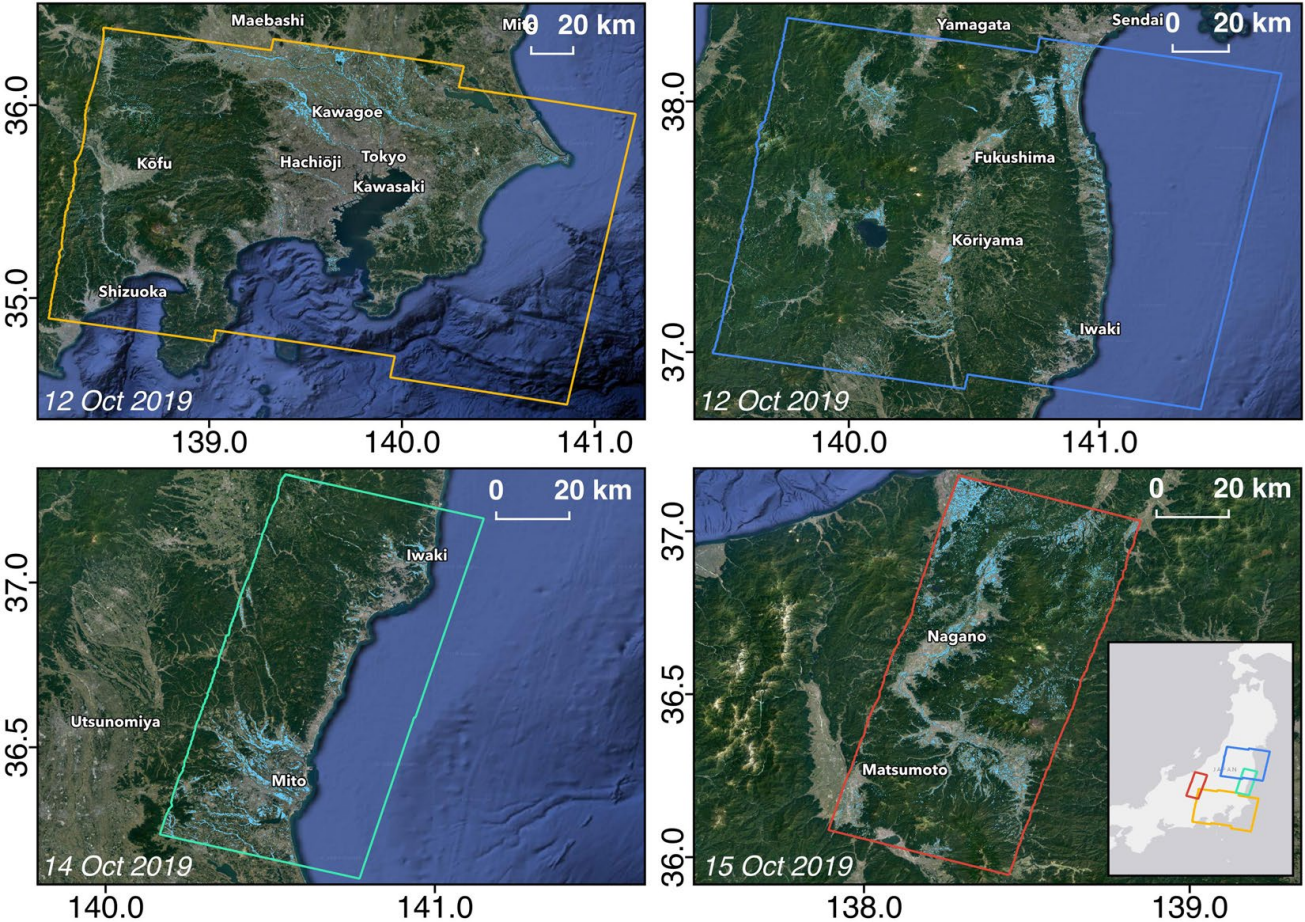
Overview of FPMs covering the Tokyo, Fukushima, Ibaraki and Nagano prefectures. Light blue pixels indicate flood, and each map extent follows the SAR footprint indicated by the colored polygons.

**Fig. 2:**
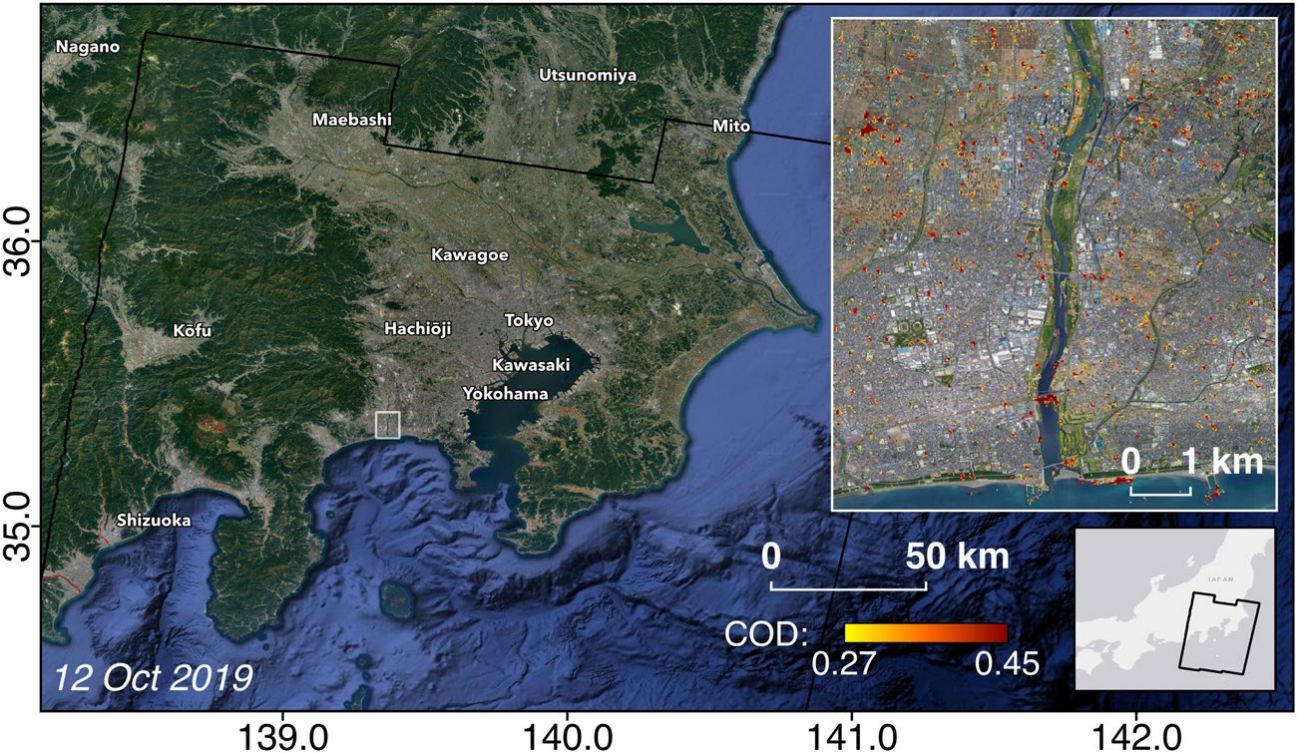
Overview of DPM covering the Tokyo prefecture. Yellow to red pixels indicate increasingly more significant land surface change, and the map extent follows the SAR footprint indicated by the black polygon. The extent of the zoomed in DPM is indicated by the white rectangle.

The Sentinel-1 SAR data we used to create our products can be obtained through the Amazon Web Services Public Dataset Program^12^. ALOS-2 data were obtained from Sentinel Asia and are not publicly available. The FPMs (Fig. 1) and DPM (Fig. 2) may be found in the ARIA-SG products^13^ (http://ariasg-products.earthobservatory.sg/EOS_ARIA-SG_201910_Japan_Typhoon_Hagibis) and ARIA share^14^ (https://aria-share.jpl.nasa.gov/201910-Typhoon_Hagibis_Japan) repositories respectively, where any updated map versions will be publicly released. Maps presented in this study are also freely provided through a static repository^15^.

The maps may be viewed through the PNG or JPEG files, or through KML files using Google Earth or GIS software. We also provide GeoTIFFs and Shapefiles of the flood and damage extents used to create the maps. These are GIS compatible and may be used for further spatial analyses. Lastly, each map has an accompanying text file detailing its usage, caveats and the SAR data it is derived from.

## Technical Validation

**Fig. 3:**
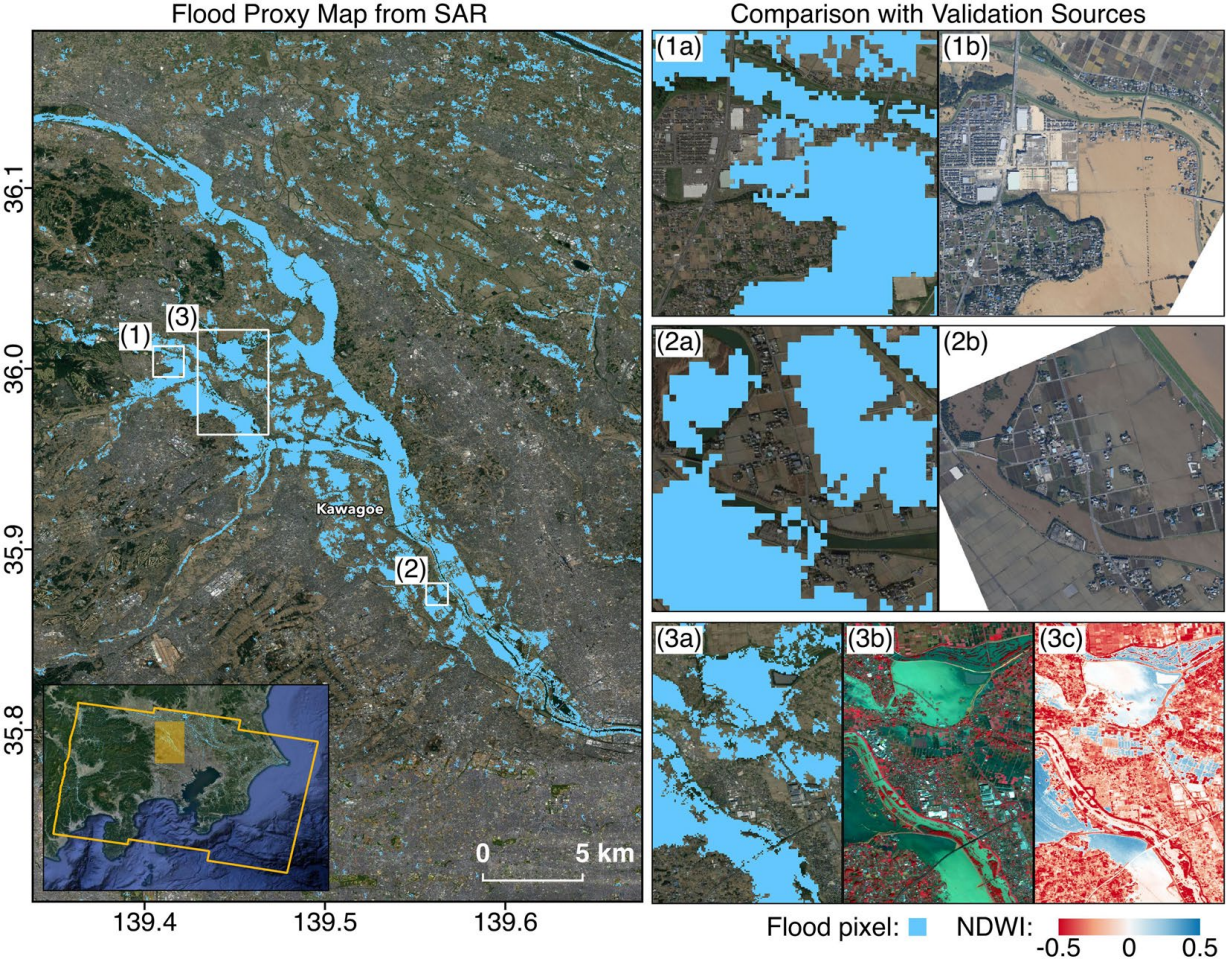
Comparison of FPM covering the Tokyo prefecture with aerial imagery from GSI and modified optical imagery from Planet. The yellow polygon indicates the SAR footprint and white rectangles indicate the extents of zoomed-in panels on the right. The FPM (12 Oct, UTC 20:43) shows agreement with aerial imagery (13 Oct, UTC 02:00) where open water floods over low-lying crop fields (**1a**,**1b**) and river banks (**2a**,**2b**), and floods from double bounce over carparks (**1a**,**1b**) were appropriately detected. The water bodies detected were also similar to those inferred from optical imagery (13 Oct, UTC 01:30) based on dark blue and cyan areas in the FCC of reflectance (**3b**) and blue areas in the NDWI (**3c**).

**Fig. 4:**
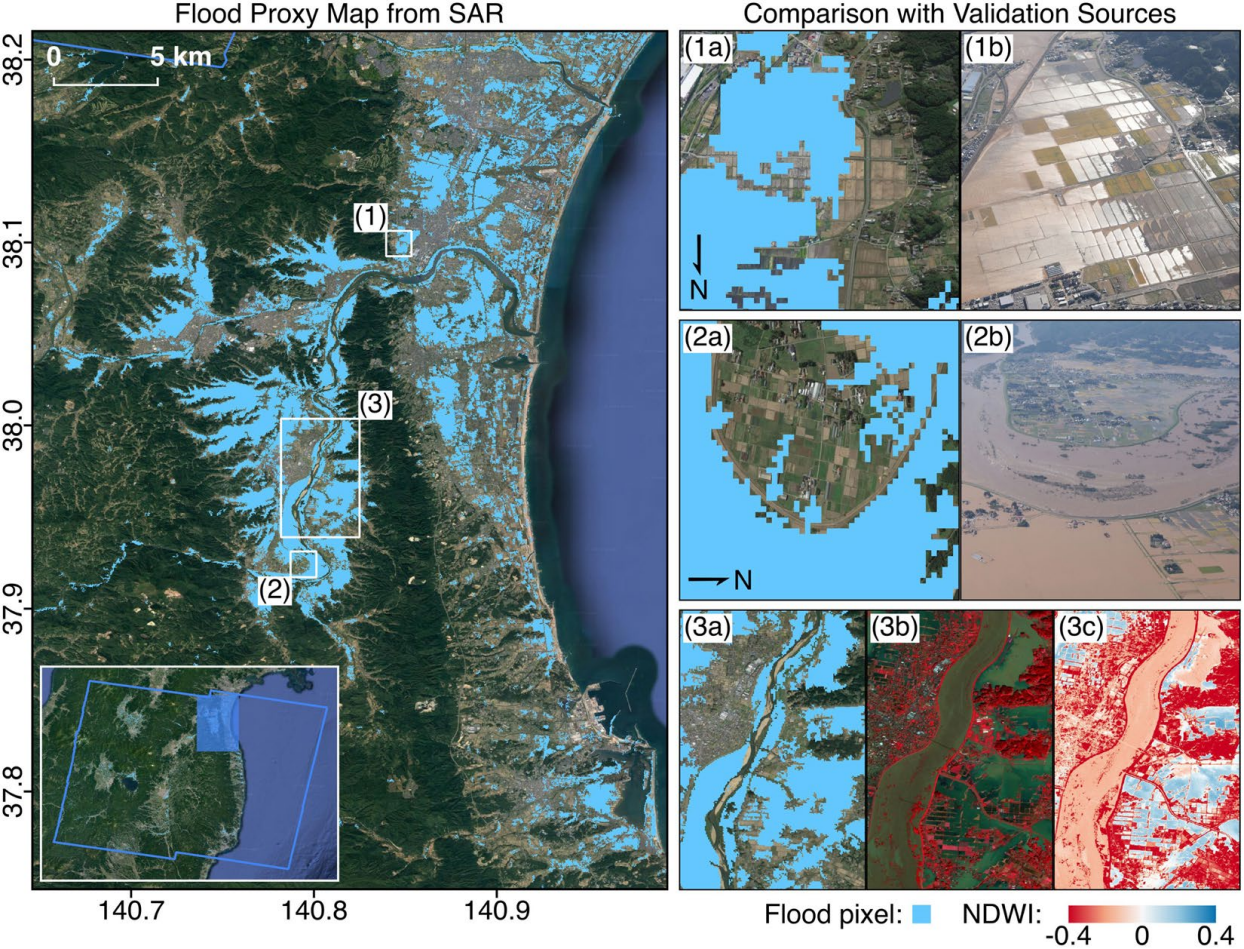
Comparison of FPM covering the Fukushima prefecture with aerial imagery from GSI and modified optical imagery from Planet. The blue polygon indicates the SAR footprint and white rectangles indicate the extents of zoomed-in panels on the right. The FPM (12 Oct, UTC 20:43) shows agreement with aerial imagery (13 Oct, UTC 04:30) where open water floods over low-lying crop fields (**1a**,**1b**) and river banks (**2a**,**2b**), and floods from double bounce next to low-rise buildings (2a, 2b) were appropriately detected. The water bodies detected were also similar to those inferred from optical imagery (13 Oct, UTC 01:22) based on dark blue and cyan areas in the FCC of reflectance (**3b**) and blue areas in the NDWI (**3c**).

During the thresholding of the SAR-based FPMs and DPM, we calibrated the thresholding with different types of data where available. Firstly, we inspected 4-band PlanetScope optical imagery^10^ of 3 m resolution (Figs. 3 and 4). Timely optical images with minimal cloud cover were obtainable, but not covering all areas of the Tokyo and Fukushima prefectures. To inspect the images, we converted digital numbers to reflectance values, and made false color composites (FCCs) of reflectance using the NIR, red and green bands, assigned to red, green and blue channels respectively^16^. In general, water has lower reflectance across all bands, separating it from the vegetation that has higher reflectance in the NIR region. Thus, the FCCs show vegetation as red, clear water as dark blue, turbid water as cyan, and built-up areas as shades of blue, yellow or grey. We also computed the normalized difference water index (NDWI) from the reflectance bands (Eq. 3)^17^.

3 $$NDWI=\frac{Green-NIR}{Green+NIR}$$

The NDWI is proportional to water content, where positive values indicate moisture and negative values indicate water stress^17^. As the NDWI is generally less reliable over turbid waters, it was inspected with the FCC of reflectance.

**Fig. 5:**
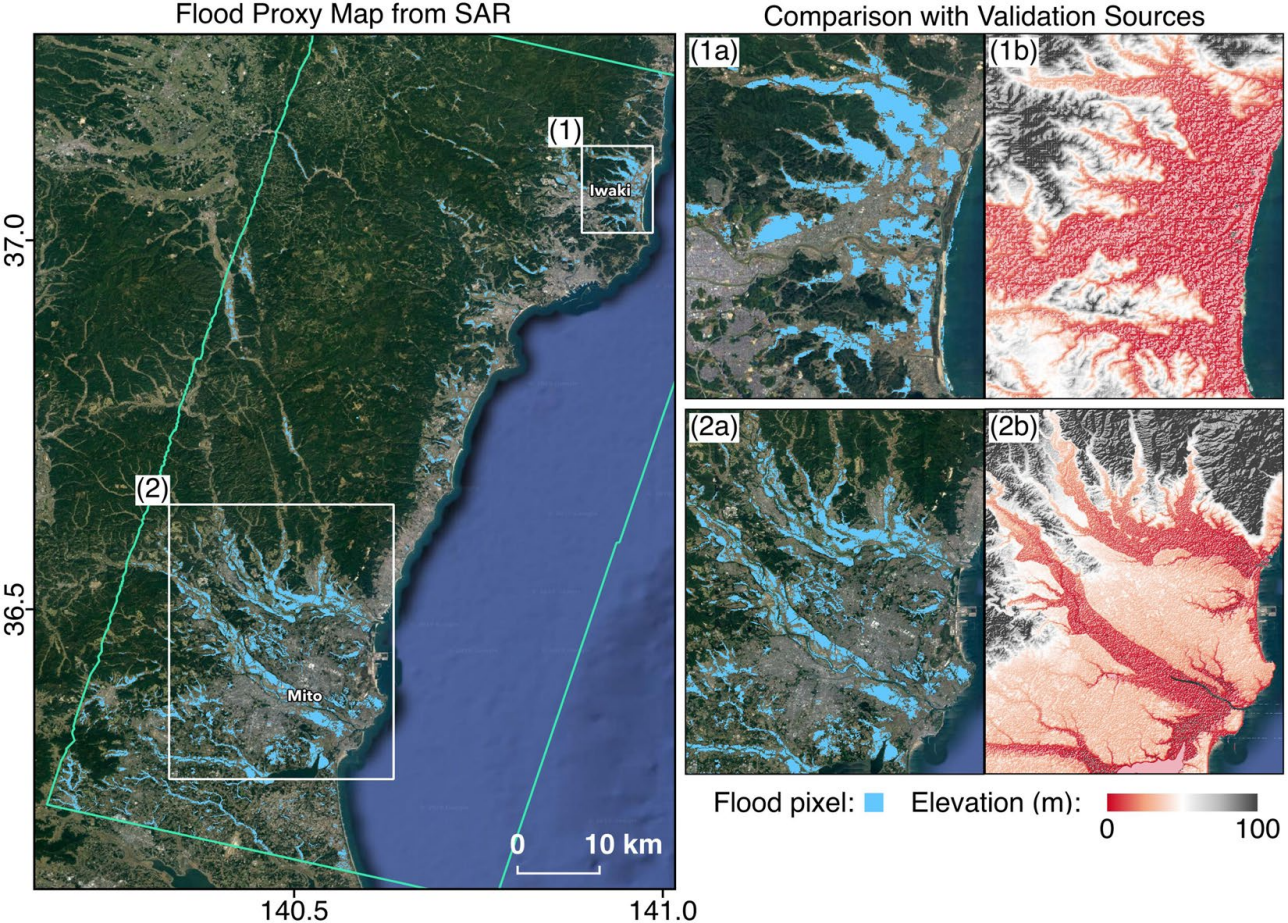
Comparison of FPM covering the Ibaraki prefecture with topography from SRTM DEM. The green polygon indicates the SAR footprint and white rectangles indicate the extents of zoomed-in panels on the right. Floods detected were confined within low-lying areas, while floods in highly urbanized areas could not be detected due to the insensitivity of amplitude changes in densely built-up areas (**1a**,**1b**,**2a**,**2b**).

**Fig. 6:**
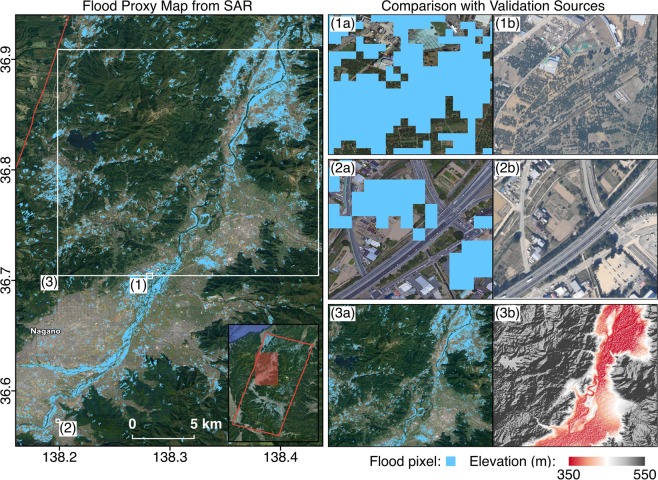
Comparison of FPM covering the Nagano prefecture with aerial imagery from GSI and topography from SRTM DEM. The red polygon indicates the SAR footprint and white rectangles indicate the extents of zoomed-in panels on the right. The FPM (15 Oct, UTC 03:37) shows agreement with aerial imagery (16 Oct, UTC 03:15) where floods from double bounce over sparsely vegetated areas (**1a**,**1b**) and next to low-rise buildings (**2a**,**2b**) were appropriately detected. Floods detected were also confined by the topography, with a higher concentration of flood waters in the lowest-lying area mapped downstream in the northeast (**3a**,**3b**).

Secondly, we used SRTM DEMs^5^ which highlight flood prone areas (Figs. 5 and 6), on the basis that water bodies tend to aggregate in low-lying areas and are confined by topographic features. Topographic data were available for all FPMs and covered whole mapping extents.

**Fig. 7:**
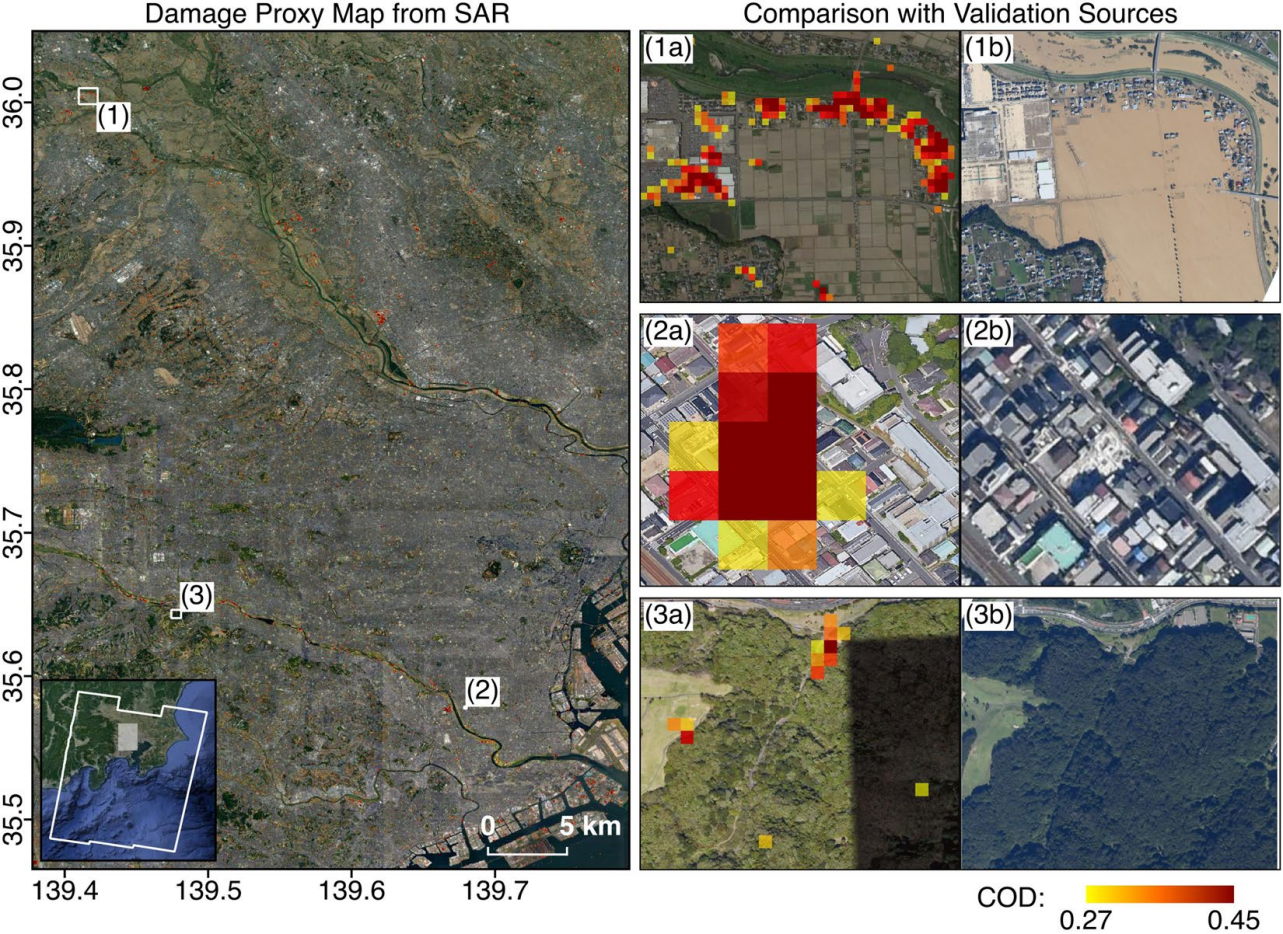
Comparison of DPM covering the Tokyo prefecture with aerial imagery from GSI. The white polygon indicates the SAR footprint and white rectangles indicate the extents of zoomed-in panels on the right. The DPM (12 Oct, UTC 20:43) shows agreement with aerial imagery (13 Oct, UTC 02:00) where decorrelation from inundated low-rise buildings (**1a**,**1b**) and sediment or debris covered roads (**2a**,**2b**) were detected. The coherence-based DPM is less reliable over naturally incoherent areas such as open water bodies (**1a**,**1b**) and vegetation (**3a**,**3b**).

Thirdly, we used high resolution aerial imagery from GSI^9^ for a visual confirmation of flooded, non-flooded, damaged and non-damaged areas (Figs. 3, 4, 6 and 7). These were available for concentrated areas in the Tokyo, Fukushima and Nagano prefectures.

Overall, fitting thresholds could be determined such that the FPMs show consistency with the validation sources over open and less built-up areas, and sparse vegetation such as crop fields. The amplitude-based mapping is less reliable over dense urbanized areas due to a large mix of backscattering components within each relatively large resolution cell, and layover and shadow effects^18^. The FPMs are also only representative of flood extents at the time of SAR acquisition and do not necessarily reflect the peak of flooding. Apart from the differential responses of the drainage systems, the peak of flooding likely correlated with reported rainfall distributions and the typhoon track as well, which both migrated from south to northeast Japan in a little less than 24 hours after the typhoon’s landfall^19^. Hence, the FPMs covering Ibaraki and Nagano are more likely past the peak of flooding.

Thresholds determined for the DPM were also fitting with validation sources over urban areas. However, the DPM may indicate inundated low-rise buildings in flooded areas and false damage pixels over vegetated areas where coherence is generally poor. The DPM should also be used as a proxy for damage extent, and may not be equivalent to conventional Post-Disaster Needs Assessments which commonly define damage as the loss of assets of monetary value. For example, cracks in building walls, water-induced damage to electrical wiring and damaged crops are unlikely detected with this SAR-based technique.

**Fig. 8:**
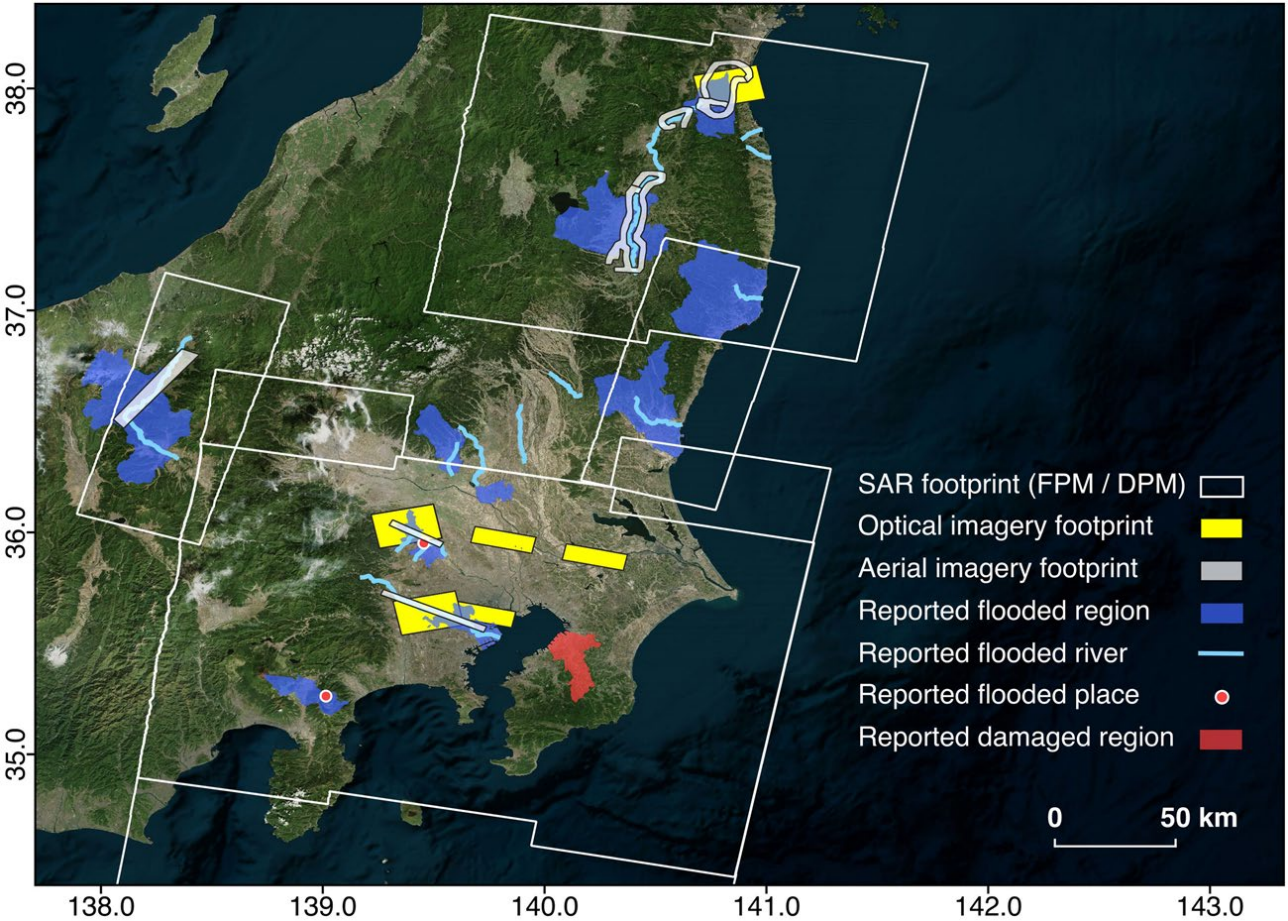
Spatial coverage of validation data used to guide thresholding, in relation to the SAR footprints of the FPMs and DPM. Reported flood and damage from news sources are non-exhaustive. Affected regions and rivers shown are broad estimates and do not refer to entire areas being flooded or damaged. We created geometries of reported flooded regions, flooded rivers and damaged regions by editing data from GSI of Japan^20^.

Uncertainties in the flood and damage extents in areas lacking validation may be present due to the global thresholding of LAR and COD values across the mapping area. This is because we assumed that flood- or damage-induced backscatter and coherence changes within a SAR footprint are more or less the same when calibrating thresholds to fit independent validation information of limited spatial coverage. Hence, higher noise levels in the mapped pixels where validation data is lacking is possible (Fig. 8). Additionally, artefacts from layover effects may be present due to a combination of the side-looking SAR satellite look angle, earth curvature, topography and buildings. Further work is ongoing to improve the reliability over different land covers and spatial variation of the maps.

## Data Availability

The codes for SAR processing using the ISCE algorithms and for generating proxy maps are available via the following GitHub repositories: https://github.com/isce-framework/isce2 and https://github.com/earthobservatory/slcp2pm.
